# Blue Power: Turning Tides into Electricity

**DOI:** 10.1289/ehp.115-a590

**Published:** 2007-12

**Authors:** David C. Holzman

Water has been used by humans as an energy source in the form of tide mills and water wheels for nearly 2,000 years. As a large-scale power source, however, tidal and wave energy is at roughly the same stage of development that wind power was at in the 1980s, numerous observers say, with many concepts but few installations—a situation that reverses as a technology matures. And the field is heating up fast, which is good news given the wealth of human and environmental health effects that follow traditional fossil fuel–fired power plants.

Tidal turbines capture the energy of the currents, as well as that of rivers, irrigation canals, dam tailraces, and possibly even ocean currents such as the Gulf Stream, in much the same manner that wind turbines transduce air currents. The diverse taxonomy of wave devices, meanwhile, could convert the ocean’s roiling into grid-ready electrons. Wave and tidal energy, known collectively as “marine energy,” is currently capable of supplying electricity equivalent to 10–25% of today’s world’s production, according to various estimates, or about 2–5% of end-use energy.

In the United States, wave energy conversion alone could supply the equivalent of 6.5% of electricity at current consumption rates, according to one fairly conservative estimate by the Electric Power Research Institute (EPRI), the research arm of the electric utility industry. This is equivalent to the electricity generated by all conventional U.S. hydroelectric plants. Tidal power could furnish another 3–3.5% of electricity needs.

In the United Kingdom, the Carbon Trust, a government-funded company promoting climate change mitigation, estimates somewhat more optimistically that in the long run, wave and tidal power together could supply 15–20% of British electricity needs. According to the 2006 Carbon Trust report *Future Marine Energy*, the United Kingdom could be using these technologies to produce two to five U.S. nuclear plants’ worth of electricity by 2020.

The zero-emission cleanliness of wave and tidal energy technologies is comparable to that of wind, and marine energy is arguably the least aesthetically disruptive method of producing electricity. Unlike the proposed Cape Wind offshore wind farm, for example, which currently has some legislators in Massachusetts saying “not in my backyard,” wave and tidal technologies are often invisible from shore.

For purposes of energy capture, water is similar to wind, except that seawater is more than 800 times denser than air, essentially making it easier to capture energy. Moreover, whereas the wind can come from any direction, in most locations the tides flow only in and out, reducing the complexity of the mechanisms required to harvest that energy.

Tidal power is readily predictable, which makes coordinating the flow of electricity in the grid quite manageable. The keys to a strong tidal current are a large rise and fall in the tides and geographical features that funnel the water through a narrow channel. As with wind, the energy available in a tidal current varies as the cube of the current’s speed. Six knots (about 6 mph) is the threshold for economic viability, according to the 2006 EPRI report *North America Tidal In-stream Energy Conversion Technology Feasibility Study*. But tides this swift are uncommon. Viable wave resources are more widely distributed.

## The Technologies

The biggest new wave project is in Portugal, where Pelamis Wave Power is building the world’s largest wave farm. In its first phase the plant will produce up to 2.25 MW, enough to power 450 average U.S. homes. The ultimate goal is 20 MW (enough for 4,000 homes). The first commercial-scale preproduction Pelamis outfit off of Scotland currently contributes up to 750 peak kW to the U.K. grid.

Like the sea snake for which it is named, the Pelamis floats atop the ocean’s surface. Each of the Pelamis converter’s segments is about the size of a train car. Passing waves bend the Pelamis at the joints. Hydraulic rams work like bicycle pumps to resist that bending, pushing oil at high pressure through hydraulic motors to drive electrical generators.

Another leading wave technology, Finavera Renewables’ AquaBuOY, sits atop a long cylinder that hangs down into the ocean. The cylinder contains a solid steel piston, sprung from each end with a hose made of steel-reinforced rubber. As the buoy bobs up and down, the heavy piston’s inertia stretches one segment of the hose while compressing the other. The potential energy thus captured, once released, pumps water through a turbine in the buoy, generating electricity. Finavera is conducting a pilot study in Washington State to be completed in 2009, with 50- and 100-MW systems eventually planned for Oregon, California, and Portugal.

Another commercial-scale system currently in operation is the 500-kW LIMPET (Land Installed Marine Powered Energy Transformer). Wavegen, its manufacturer, installed this shoreline energy converter on an island off of Scotland in 2000. The LIMPET shunts incoming waves into a shore-mounted container. The “oscillating water column” within forces air back and forth through a turbine, driving a generator.

There are 25 concepts for capturing energy from tidal currents. One of the leaders is the tidal turbine manufactured by OpenHydro Group, which was chosen by Nova Scotia Power for projects in the exceptionally harsh but potentially abundant reservoir of energy that is the Bay of Fundy. The bay is home to some of the world’s strongest tides, rising and falling nearly 50 feet, and its Minas Passage has the fastest tides in the world at 8 knots, says Margaret Murphy, manager of public affairs for Nova Scotia Power. That speed is both good and worrisome. “Picture a ton of ice, encrusted with sand, moving at eight knots, [and crashing into the turbine],” she says.

Nova Scotia Power plans to begin testing a 1-MW OpenHydro unit in the Minas Passage starting in late 2009. “The eventual dream would be to deploy three hundred megawatts in this one passage,” Murphy says—enough for an average capacity of around 170 MW.

Another tidal generator, manufactured by Ocean Renewable Power Company, has the advantage of being able to continue reaping tidal energy without any mechanical repositioning as the tide shifts. The blades of these large turbines—two of which fit into a tractor trailer–sized module with a generator between them—trace the outline of a cylinder as they rotate, rather than a circle. Their spinning turns a shaft at the central axis of the cylinders.

The company hopes to develop tidal farms in Maine, Alaska, and possibly the Gulf Stream, although the latter’s distance from shore currently renders it economically marginal for less than a gigantic farm. The company’s Maine subsidiary and the city of Eastport plan to have a full-scale prototype operating early in 2009, with 40 MW of commercial power planned for the state and more installed elsewhere.

Perhaps the most radical concept in tidal power is the use of vortex-induced vibrations—the same phenomenon that toppled the Tacoma Narrows Bridge in 1940—to generate power. Vortex-induced vibrations occur whenever a current flows around a flexible cylindrical structure. Vortices are shed sequentially from alternate sides of the cylinder, causing the cylinder to oscillate. A tidal current device dubbed VIVACE (Vortex Induced Vibrations for Aquatic Clean Energy) transduces that oscillation into electricity.

This approach, if proven successful, could greatly expand the tidal and ocean/river resource because it can harvest ample power from the vast majority of currents that fall well below the EPRI economic viability threshold of 6 knots. Currently, the engineering firm Vortex Hydro Energy is developing a pilot project to generate 3 kW from a current of less than 2 knots on the Detroit River for the Detroit Power Authority, with the power to be used to light a wharf.

## Costs and Benefits

At this early stage in development, electricity from wave and tidal generators is not inexpensive. Wavegen is peddling its wares to Pacific Island nations, where the cost of wave power—40–50 cents per kWh—competes with local diesel generators. In Scotland, the cost is 18–21 cents per kWh, says Dave Gibb, Wavegen’s general manager. By comparison, in the mainland United States, the per-kWh cost of electricity averages around 7–8 cents, but can reach 20 cents in some regions. Roger Bedard, the ocean energy leader at the EPRI, notes that wind started out around 40 cents per kWh, and has declined to around 7 cents. He expects wave and tidal costs to drop similarly, eventually costing even less than wind.

Marine energy is notably environmentally benign. The 2004 EPRI report *Offshore Wave Power in the US: Environmental Issues* states that “given proper care in site planning and early dialogue with local stakeholders, offshore wave power promises to be one of the most environmentally benign electrical generation technologies.”

Like hydroelectric dams, wave and tidal technologies are nonpolluting. But unlike dams, which block whole rivers, tidal turbines do not require water impoundments nor do they appear to interfere with migration of fish or other animals or otherwise interfere with the ecology. A study by Oak Ridge National Laboratory published in the October 2005 issue of *Hydro Review* placed the probability of migrating fish being injured by the tidal turbine project in New York City’s East River at 0.004–0.457%.

“I haven’t heard of any specific environmental concerns with [wave and tidal power] yet, but it’s something we will continue to follow,” says John H. Rogers, senior energy analyst and Northeast Clean Energy Project manager at the Union of Concerned Scientists. Rogers asserts that any environmental impact must be balanced against the impact of forgoing these technologies—for example, the construction of more coal or other fossil fuel plants, with their attendant environmental impacts.

Nonetheless, some genuine environmental concerns need to be studied, says Keely Wachs, environmental communications manager at Pacific Gas and Electric, which is aggressively pursuing renewables. Along the West Coast, he says, the majority of gray whale migration routes run within 2 nautical miles of shore, coinciding roughly with the likely siting of wave power plants relative to shore. Installing such devices could destroy the kelp forests where migrating mother–calf pairs shelter. Wave machines could also conceivably interfere with local fishing industries, says Wachs. Again, however, careful siting could obviate many if not all these concerns.

## Regulating the Tides

About one-third of U.S. states have production goals and incentives for renewable energy, including marine energy. One of the most ambitious states, Oregon, has set a goal of 25% renewable electricity by 2025. Oregon also provides a state tax credit of 50% per installation, up to $20 million, as well as low-interest loans for wave and tidal projects.

The European Union is pursuing wave and tidal energy even more aggressively. Whereas the United States offers federal subsidies for renewable electricity of 1.9 cent per kWh, Portugal, the most generous of the European countries, offers nearly 30 cents per kWh.

In the United States, the biggest roadblock to swift adoption of wave and tidal technologies is regulation. It takes around five years and millions of dollars’ worth of studies to gain permission to plant a prototype turbine in a tidal current or in offshore waves. “It took us more than four years to get approval to put six turbines in the water,” says Trey Taylor, president of Verdant Power, which is testing its tidal turbines in the East River. During those four years, “we had to keep the engineering team together, and pay salaries.” The cost of permitting alone was more than 50% of the total project costs to date.

The problem is that there are few baseline environmental impact data “since none of these projects have been deployed before,” says Mary McCann, a fish biologist who is manager of environmental services at Devine Tarbell and Associates, a consulting firm in Portland, Maine. “How do you get projects in the water to collect information to answer these questions when you are supposed to have the answers first, in order to get approval to put them in the water?”

“A sense of proportionality needs to be built into the process,” says Sean O’Neill, president of the Ocean Renewable Energy Coalition, the national trade association for marine renewable energy. “Treating these technologies as though they are utility-scale projects is causing companies like [Verdant] to devote the majority of their capital on permitting instead of new technology development.”

The problem is not so much with the Federal Energy Regulatory Commission, which regulates licensing, as with all the other agencies that get their say, says McCann. Furthermore, says Tim Oakes, a senior regulatory advisor with Kleinschmidt Energy and Water Resource Consultants in Strasburg, Pennsylvania, wave and tidal projects frequently are held to higher standards than conventional power projects, because they are located in waterways and offshore, thus falling under federal regulatory jurisdiction. In contrast, he says, conventional power plants are usually subject to the much milder state regulation.

McCann believes the federal government should provide assistance and research funds for wave and tidal development, with the information gained to be shared with all. Although there is currently no wave or tidal energy program within the U.S. DOE, legislative efforts to establish such are flowering across Capitol Hill, says Walter Musial, a senior engineer at the DOE National Renewable Energy Laboratory.

Some of these problems may soon be resolved, says Oaks. On 2 October 2007, the Federal Energy Regulatory Commission held a workshop in Portland, Oregon, where it proposed a program to complete licensing for several types of pilot projects within six months.

## Figures and Tables

**Figure:**
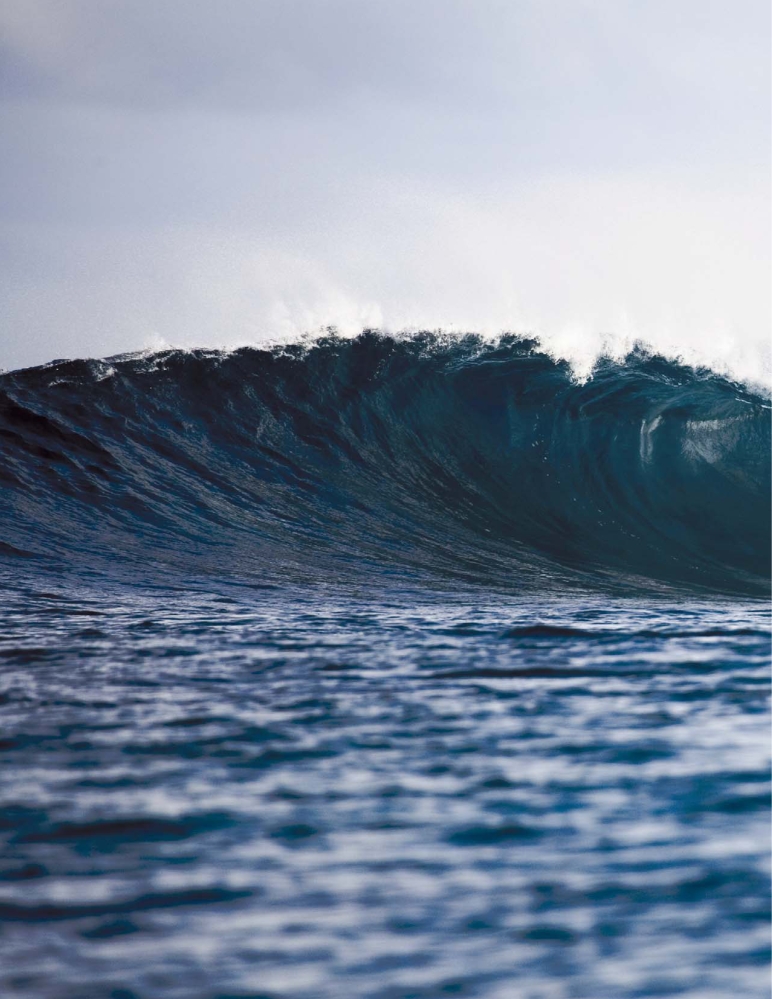


**Figure:**
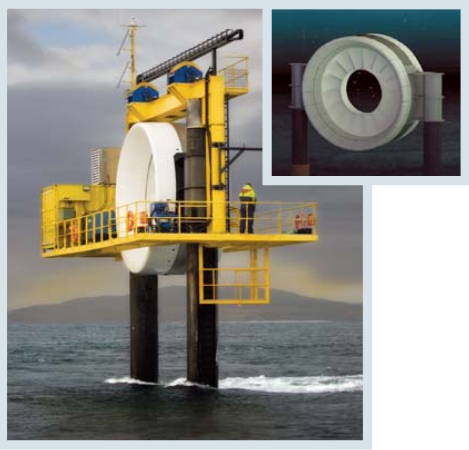
OpenHydro tidal turbine

**Figure:**
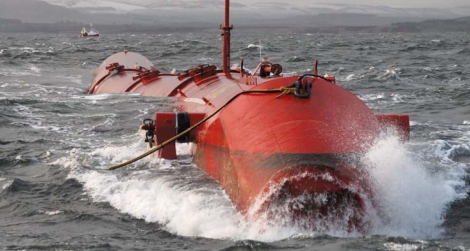
Pelamis wave converter

**Figure:**
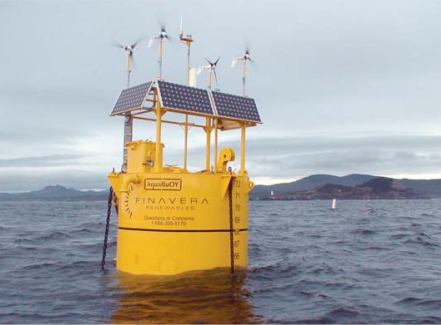
AquaBuOY

**Figure:**
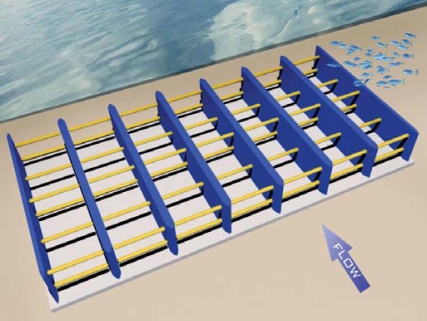
VIVACE device
